# Cutting the research pie: a value-weighting approach to explore perceptions about psychosocial research priorities for adults with haematological cancers

**DOI:** 10.1111/j.1365-2354.2010.01188.x

**Published:** 2011-05

**Authors:** CL Paul, R Sanson-Fisher, HE Douglas, T Clinton-Mcharg, A Williamson, D Barker

**Affiliations:** School of Medicine and Public Health, Health Behaviour Research Group, University of NewcastleCallaghan, New South Wales; The Leukaemia Foundation of AustraliaWindsor, Queensland; School of Medicine and Public Health, Centre for Epidemiology and Biostatistics, University of NewcastleCallaghan, New South Wales, Australia

**Keywords:** blood cancer, research priorities, consensus methods, consumer groups, psychosocial research

## Abstract

Despite the burden of illness associated with haematological cancers, little research is available about improving psychosocial outcomes for this group. Given scarce research funds, it is important to ensure that resources are used strategically for improving their psychosocial well-being. This study aimed to identify the perceptions of professionals, patients and carers regarding prioritising psychosocial research efforts. First, an expert panel's views on priorities for research were identified. This was followed by a web survey to obtain the perceptions of 117 health professionals, patients and carers. The value-weighting survey used points allocation, allowing respondents to indicate the relative priority of each option. A substantial proportion of resources were allocated to patients who were newly diagnosed or receiving treatment. Less priority was given to other stages of the cancer journey or non-patient populations. There was no indication that any type of psychosocial research was a priority; however, some differences were identified when comparing the priorities of the three respondent groups. To improve psychosocial outcomes for haematological cancer patients, resources should be directed towards patients in the early stages of the cancer journey. There may be a need for research investigating potential interventions to improve psychosocial outcomes for patients with haematological cancers.

## INTRODUCTION

### Haematological cancers are associated with a high burden of illness

As treatment outcomes improve and the population ages, haematological cancers such as lymphoma, leukaemia and myeloma represent a growing cancer survivor population (Australian Institute of Health and Welfare (AIHW) & the Australasian Association of Cancer Registries (AACR) 2008). In countries such as Australia, haematological cancers such as lymphoma and leukaemia represent the 6th and 8th most common cancers (Australian Institute of Health and Welfare (AIHW) & the Australasian Association of Cancer Registries (AACR) 2008). However, survival is relatively poor (Australian Institute of Health and Welfare (AIHW) & the Australasian Association of Cancer Registries (AACR) 2008), and treatments for these cancers can result in a range of debilitating side effects (Richardson *et al*. 1988).

### The importance of psychosocial health for people with haematological cancers

Rates of clinical distress have been reported to range between 32% and 48% for haematological patients (Carlson *et al*. 2004). Treatment can have a devastating impact on the lives of those diagnosed and treated, affecting fertility, sexuality (Schover 2005) and the ability to fulfil usual vocational, social and family roles (Sherman *et al*. 2005). Despite this, psychosocial issues among people with haematological cancers and their support persons remain relatively unstudied compared with survivors of other common types of cancer such as breast, prostate and colorectal cancers (Macvean *et al*. 2007).

### A need for targeted research into improving psychosocial health

A range of supportive interventions including screening for psychosocial symptoms have been trialled with some cancer survivor groups with limited success (McLachlan *et al*. 2001; Aranda *et al*. 2006; Boyes *et al*. 2006). While research suggests such interventions may help, the evidence base regarding how to improve psychosocial outcomes for cancer survivors is in its infancy (Aziz 2007). Funds available for such research are also quite limited. Adult haematological cancers in particular may attract relatively little funding due to a lower community profile than other groups. Short survival times (Australian Institute of Health and Welfare (AIHW) & the Australasian Association of Cancer Registries (AACR) 2008) also limit the opportunities for rigorous intervention studies with this group. Consequently, it is important that the available research resources are used strategically to address key issues.

Both researchers who choose the focus of their research efforts and those involved in making decisions about strategic directions for research funding would benefit from an understanding of what kinds of psychosocial research are most needed. Research with other cancer groups (Grassi & Rosti 1996; Devine 2003; Hodgkinson *et al*. 2007) suggests it is important to consider which populations should be the focus of research as well as the research topic or type. Populations that should be considered include patients at various stages of survivorship and non-patient groups such as partners or carers. Psychosocial research topics may range from the consideration of measurement issues (Thewes *et al*. 2004), to evaluation of specific therapies (Marcus *et al*. 1998) or changes in the treatment environment (Mills *et al*. 2009).

### Who should make decisions about where to target research funding?

In the absence of data, the first step in devising a strategic psychosocial research agenda is to seek the views of a wide range of relevant ‘experts’ about which research is most needed to deliver the greatest benefit. The term ‘expert’ should not be limited to those with the highest level of clinical expertise or those able to allocate funding. The growing emphasis on consumer involvement in decision making (Fink *et al*. 1984; Oliver *et al*. 2004) and a multidisciplinary approach to cancer care (Ko & Chaudry 2002) necessitates recognition that each group is an expert in its own area of endeavour. Therefore, deciding on a strategic research agenda for improving psychosocial outcomes should involve patients, their carers, clinicians, researchers, social workers, psychologists and patient advocates. While the views of these various experts may not always agree, each perspective is valid.

### What method should be used to make decisions about psychosocial research priorities regarding haematological cancers?

Any funding agency with limited resources has to make a decision about where to spend its money. There are a range of methods for determining research priorities including advocacy-based decision-making approaches that take into consideration the views of individuals or small groups of experts or stakeholders who advocate for research in a particular area (Chalkidou *et al*. 2009). In contrast, consensus-based approaches such as the Delphi process do not rely on individual stakeholder perceptions alone, but rather aim to determine the level of agreement between stakeholders (Beretta 1996). Funding decisions require some sense of the perceived relative value of the options. Concepts from the field of health economics such as ‘willingness to pay’ and techniques such as the ‘standard gamble’ (Shiell *et al*. 2002) can assist with this need. However, these techniques often place a high demand on the respondent if the relative value of a large range of options must be determined. An alternative approach is to provide experts with a scenario similar to that faced by decision makers – what proportion of funding should be directed towards each of the available research options?

### An alternative method for obtaining perceived research priorities

One such method is the two-step value-weighting approach, which is designed to capture breadth and difference of opinion in a cost- and time-efficient manner, increasing the likelihood of its being regularly used. The first step involves using a modified Delphi approach to develop a list of potential priority research items. Just as the Delphi method recognises the importance of collecting and distilling the knowledge of experts (Adler & Ziglio 1996), the value-weighting approach assumes that the collective wisdom of a broadly constituted group of experts (professionals and consumers) is a key first step in defining a list of potential research options. The high degree of commitment required by the repeated rounds of a Delphi process (McKnight *et al*. 1991) may preclude the involvement of some participants such as patients undergoing treatment. A modified process that limits the role of the Delphi group to constructing a list of strategies without rating their relative importance has the capacity to reduce participant response costs and time required. The second step in the value-weighting process involves a resource allocation (value-weighting) exercise in the form of a survey with the wider expert population. This second step allows a large number of participants to anonymously indicate the relative perceived value of each item in a timely manner. Underpinning the resource allocation exercise is the recognition that a complex interplay of factors must be distilled into single decisions when making strategic plans for research. These complex considerations must then be applied in the context of a limited funding base. This method mimics the decisions often faced by those who need to allocate resources for research or programme delivery. By requiring a range of participants to frame their decision in terms similar to that faced by the funding agency itself, the direct relevance and potential usefulness of the data is increased. The views of participants can be either aggregated to obtain an overall view or separated by participant group to explore whether, e.g. clinicians and patients differ in their perceived priorities for research. Therefore, the value-weighting method is a time-efficient way to open up opportunities for increased breadth and depth of participation, potentially increasing the likelihood that funding agencies will take a structured approach to allocating research priorities.

### Aims

This study will explore the perceived psychosocial research priorities for older adults (aged 30 years or older) with haematological cancers from the perspective of health professionals, patients and partners/carers. Participant perceptions regarding the relative benefit associated with research efforts will be analysed in light of:

1. Research populations: e.g. patients undergoing treatment, patients who have relapsed or carers.
2. Psychosocial research: e.g. describing which patients are at high risk of poor psychosocial outcomes versus testing the effectiveness of particular forms of therapy or support.
3. Respondent groups: e.g. comparing the priorities of each respondent group to determine whether there are differences in views about research priorities.

## METHODS

A two-stage value-weighting process was used, first to identify the range of potential options for psychosocial research, how these might be presented in a survey format (stage I) and then to quantify the potentially different views of a varied group of health professionals, patients and carers on this topic (stage II).

### Methodological strengths

The strengths of the current methodology arise from both the sampling method and the modified Delphi approach used to construct the survey. Although the current sample was made of both self-selecting and convenient samples, it encompasses a broad group of stakeholders who are likely to be most affected by research funding allocation. While clinicians are often over-represented in comparison with patients in decision-making processes (Oliver *et al*. 2004), the value-weighting approach facilitates the involvement of a more balanced proportion of healthcare providers and consumers. The inclusion of a value-weighting survey has the flexibility to determine whether and where there is consensus in a timely manner.

### Stage I

A national advisory group consisting of leading researchers, healthcare professionals and patient representatives in the areas of blood cancer and psychosocial outcomes was formed under the auspices of the Leukaemia Foundation of Australia (The Foundation). The Foundation nominated 13 individuals to represent a variety of stakeholders and viewpoints including consumers, clinicians, providers of psychosocial care, researchers and those involved in programme management and advocacy. In-principle definitions of expertise were used including peer recognition and previous involvement in consumer-led activities (consumers), number of years of experience (clinicians and programme managers), research track record (researchers) and degree of patient contact (providers of psychosocial care). The advisory group were involved in three rounds of a modified Delphi via teleconferences and email communications to develop a list of potential priority areas for psychosocial research in conjunction with the authors. The task of the group was to achieve consensus regarding the range of research topics to be included in the web survey. The multiple rounds permitted a reduction on the complexity and number of potential items presented to participants in the web-based survey.

The outputs of stage I were:

- A list and definitions of population groups/steps in the cancer journey.
- A list and definitions of types of psychosocial research.
- Question stems and response scales for the web survey (described in stage II below).

### Stage II

#### Sample

The advisory group nominated the names of Australian experts in their field to form the survey sample. This included clinicians, researchers, psychosocial care providers such as psychologists or social workers, and those involved in programme development or delivery in the area of psychosocial care. This group is described as ‘health professionals’ for brevity from here onwards.

The Foundation promoted the survey via its regular newsletter to patients and carers. Patients could include those at any stage of care. Carers could include any partners, family members or friends who had provided any form of care to a patient with haematological cancer. Those interested in completing the survey and with Internet access responded by email to the researchers.

#### Procedure

All potential web survey participants (health professionals, patients and carers) were provided with a unique web link to the survey in November 2008. Up to two email reminders were provided to those who had indicated their interest and did not complete the survey within 4 weeks of the initial email.

#### Web-based survey

The survey provided a value-weighting scenario to allocate token funding to different areas of psychosocial research to show which research should be given priority: ‘Imagine that the Leukaemia Foundation has asked you to decide which areas of psychosocial research should be funded. How would you allocate funding among these research areas so that they have the greatest impact on psychosocial outcomes for older adults diagnosed with blood cancer and their families? You can allocate all the funding to one area, or allocate different amounts of funding to different areas.’

#### Research populations

Each participant was asked to allocate 100 points of ‘funding’ across the following research populations/cancer stages:

- Patients newly diagnosed or in treatment.
- Patients finished initial treatment or receiving maintenance treatment.
- Patients who have relapsed.
- Patients receiving palliative care.
- Partners and carers.
- Other family or friends.

#### Psychosocial research

Participants then allocated another 100 points of funding across the following types of psychosocial research:

- Developing measures to identify psychosocial concerns.
- Identifying who is at risk of poor psychosocial health and who is resilient.
- Testing the benefit of providing better information and education for all.
- Evaluating the effectiveness of physical or psychological therapies for those who need it.
- Testing the benefit of improving social, community and spiritual support options.
- Testing the benefit of improvements in treatment centres or care delivery.
- Incorporating research into practice.

Each survey item was presented with an ‘i’ icon that allowed the respondent to view the more detailed definitions for each item.

#### Respondent groups

Participants also completed items on demographic characteristics (age, gender), and where relevant: disease characteristics (disease type, time since diagnosis, stage of care, treatment received), carer/support person details (disease characteristics of person cared for) or professional background (field of speciality and primary role, research position and areas of research).

#### Analysis

Data collected from the web-based survey was analysed in STATA (Boston & Sumner 2003) using descriptive statistics. Histograms and frequency distributions revealed that: distribution of funding allocation was normal or near-normal for all response categories. Therefore, the mean allocation was used to rank the perceived importance of the research populations and types. The means and 95% confidence intervals for each research population and psychosocial research type were calculated and plotted against a line representing non-preferential funding allocation. Two-tailed non-parametric tests (Kruskall–Wallis) were used to assess whether the respondent groups differed from each other in their mean research allocations for each item, using an alpha level of 0.05.

The University of Newcastle Human Research Ethics Committee provided ethical approval for this study. Participants provided their implied informed consent by completing the survey.

## RESULTS

### Survey sample

A total of 191 potential participants were identified and a total of 117 web surveys were completed (an overall response rate of 61%), with 41 participants describing themselves as health professionals (4 clinicians, 23 nurses, 11 allied health workers, 3 other), 45 as patients (26 were diagnosed with leukaemia, 6 with multiple myeloma, 10 with lymphoma and 7 with other forms of blood cancer) and 31 indicating they were carers. The majority of patients had received their diagnosis more than 1 year prior to completing the survey (*n*= 42, 93%). Over 50% of patients reported being finished treatment and having check-ups (*n*= 23, 51.1%). Only six patients were currently in the curative treatment stage (13.3%), with none reporting being in palliative care. Health professionals were of an average age of 40.9 years old, survivors 57.3 years and carers 55.9 years. The proportion of women in each group was 47% for health professionals, 90% for survivors and 65% for carers.

### Perceived priorities for research populations

The overall perceived importance of research with each population group in terms of improving psychosocial outcomes for blood cancer patients aged 30 years and older is described in Table 1. Mean research allocation is presented in rank order from highest proportional allocation to lowest allocation. The mean allocation to each research population varied from the highest perceived priority (29% of research allocation) for patients newly diagnosed or in treatment, down to the lowest priority being family and friends other than partners or carers (4.8% of research allocation).

**Table 1:** Mean proportion and rank for research allocated for research population and type of psychosocial research

	Mean research allocation (%)				
		
	Healthcare professionals (*n*= 41)	Patients (*n*= 45)	Carers (*n*= 31)	All (*n*= 117)	Overall rank (*n*)
Research population					
Patients newly diagnosed or in treatment	25.4	30.7	30.3	28.8	1
Patients who have relapsed	23.5	21.9	18.1	21.5	2
Patients finished initial treatment or in maintenance treatment	20.9	16.7	17.2	18.3	3
Partners and carers	14.8	13.2	14.0	14.3	4
Patients receiving palliative care	10.0	13.6	15.0	12.4	5
Other family and friends	5.3	3.8	5.5	4.8	6
Type of psychosocial research					
Develop measures to identify psychosocial concerns	12.9	17.1	16.1	15.4	1
Evaluate effectiveness of physical or psychological therapies for those who need it	20.0	15.9	12.9	16.6	2
Test the benefit of improving treatment centres or care delivery	15.4	15.3	17.7	16.0	3
Identify who is at risk of poor psychosocial health and who is resilient	14.3	17.8	12.1	15.1	4
Incorporating existing research into practice	13.6	13.4	15.3	14.0	5
Testing the benefit of providing better information and education for all	12.8	10.5	14.2	12.3	6
Testing the benefit of improving social, community and spiritual support options	11.1	9.9	11.6	10.8	7

**Figure 1:**
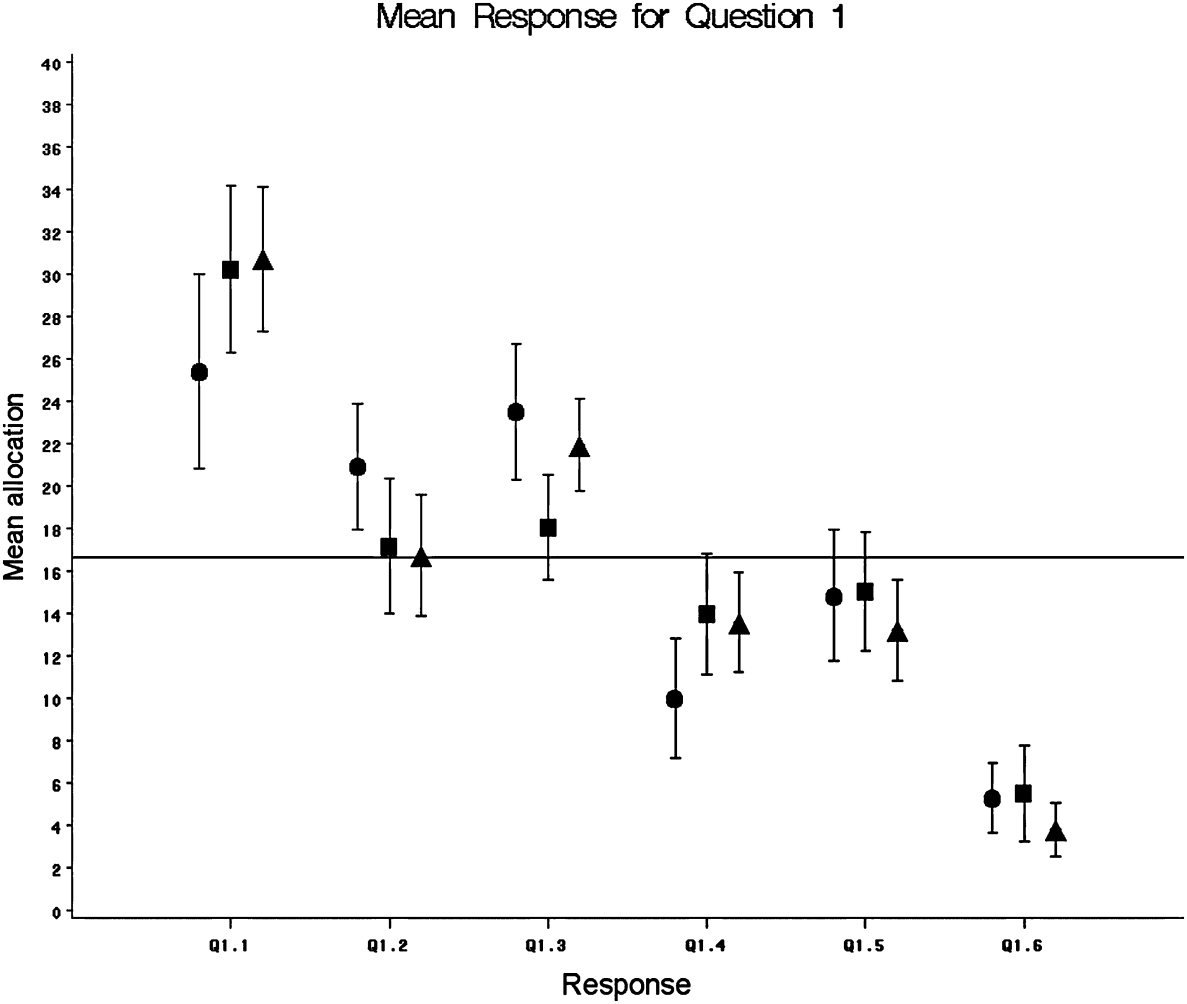
Allocations to research populations according to participant group. Labels: 1.1 = newly diagnosed patients, 1.2 = patients in maintenance, 1.3 = patients in relapse, 1.4 = patients in palliative care, 1.5 = partners and carers, 1.6 = other family and friends. (•) provider; (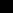) carer; (▴) patient.

Plotting the mean research allocations and 95% confidence intervals against a line representing non-preferential funding allocation (i.e. allocating 16.67 points to each of the six research populations) provides an indication of the variability among individuals' research allocations (confidence intervals) and whether the allocations appear to be significantly different from each other. As shown in Figure 1, all participant groups gave significantly higher priority to psychosocial research directed towards patients who are newly diagnosed or in the active phases of treatment. Psychosocial research relating to other family and friends was seen as a significantly lower priority than any of the other populations.

### Perceived priorities for psychosocial research

**Figure 2:**
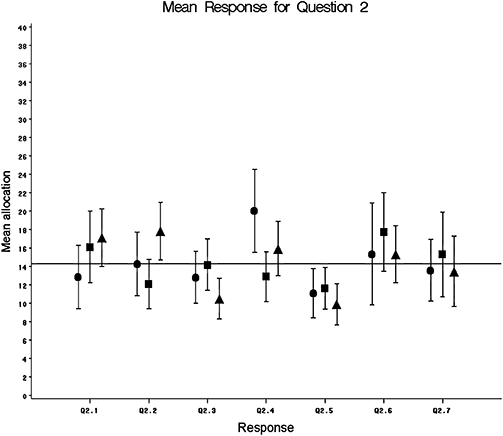
Allocations to psychosocial research areas according to participant group. Labels: 2.1 = develop measures, 2.2 = identifying who is at risk, 2.3 = test education, 2.4 = evaluate psychological therapies, 2.5 = test social and community support, 2.6 = treatment centre, 2.7 = research into practice. (•) provider; (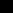) carer; (▴) patient.

The overall perceived importance of each type of psychosocial research is described in Table 1. The mean allocation to each research type varied from the highest perceived priority being the development of measures to identify psychosocial concerns (15% of allocation), down to the lowest priority being testing the benefit of improving social, community and spiritual support options (11% of research allocation). As shown in Figure 2, the overall allocations to research types were generally not significantly different from non-preferential allocation (i.e. allocating 14.28 points to each of the seven options).

### Agreement between respondent groups

In the case of research populations, a similar pattern of preferences appeared for each respondent group (see Table 1). The Kruskall–Wallis test indicated that there were significant differences in mean allocation by participant group only in terms of provider allocations to the relapsed population groups being significantly higher than were the allocations of carers (*P*= 0.02).

In the case of psychosocial research types, patient allocations to the identification of those at risk of poor psychosocial health were significantly higher than were carer allocations to this type of research (*P*= 0.04). The difference between carer and provider allocations to evaluate the effectiveness of physical or psychological therapies was also significant (*P*= 0.02), with healthcare providers providing a greater allocation than partners/carers.

## DISCUSSION

### Perceived priorities regarding research populations

For all participants combined, greater psychosocial research priority was apportioned to patient populations in the earlier stages of haematological cancer – being newly diagnosed or in treatment. It should be noted that while relatively small allocations (approximately 14%) were directed towards partners/carers and patients receiving palliative care; these areas were still considered worthy of research, albeit with lower priority. It may be worth exploring why a relatively low perceived priority was evident for research into palliative care, given that need for psychosocial support may be high in this phase. It is possible that the sample does not contain enough participants with a need for or expertise in palliative care to fully represent this aspect of the cancer journey. Despite the low perceived priority for research on this topic, the little available research does not suggest that palliative care for haematological cancers operates at an optimal level (McGrath 2002).

The results present a reasonably clear case for strategic allocation of research resources to those who are newly diagnosed or receiving treatment. These findings appear to be in contrast to the ethical constraints often faced by researchers, where gatekeepers such as clinicians or ethics committees prohibit research access to patients in these vulnerable stages. Research protocols that facilitate sensitive approaches to such patients and allow for research rigour may be necessary.

### Perceived priorities regarding psychosocial research types

Study participants did not appear to have a strong preference for one type of research over another, with one exception: in the case of the provider participant group, there appeared to be a preference for evaluating the effectiveness of physical or psychological therapies for those who need it (20% of research allocation). This may indicate that healthcare professionals, who are in the position of providing referrals for distressed patients or directly providing psychosocial care, may feel there is a need for more data in order to provide evidence-based care. Reviews of the literature support the need for more rigorous evidence on the effectiveness of interventions for psychosocial health in cancer patients (Trivedi *et al*. 2007; Boesen & Johansen 2008; Ranmal *et al*. 2008; Goedendorp *et al*. 2009).

There are a number of potential interpretations of the apparent overall lack of discrimination regarding priorities across the different types of research. First, it may be that there is an evident need for research in all of these areas, and each is perceived to have similar potential for improving psychosocial outcomes for people with haematological cancers. Second, it may be that the participants did not feel well-informed about the range of research types they were asked to consider. Third, the level of complexity involved in comparing quite different types of research (e.g. the development of measures versus testing the benefit of improved treatment centres) limited participants' ability to differentiate between items. In the absence of a more in-depth examination of reasons for these allocations, the data presented here suggest that funding should be directed towards a range of types of psychosocial research interventions for haematological cancer patients.

### Agreement between respondent groups

The allocations provided by each of the participant groups suggests that in general terms there was compatibility in the preferences expressed by health professionals, patients and carers. There was a strong correspondence in the relative rankings for both research populations and types of research even though each subgroup gave slightly differing weights or allocations in some instances. This may reflect a general need for research in this field. Providers gave a higher priority to patients experiencing relapse and to evaluating psychological therapies than non-provider groups, while patients placed a greater emphasis on the identification of patients at risk of experiencing psychosocial morbidity. This may be because patients feel that psychosocial problems are not readily identified by the health system. This is not surprising, given the data suggesting that clinicians are not always good at identifying patients who are experiencing psychosocial problems (Trivedi *et al*. 2007). However, the relative consistency of results across groups suggests that the growing impetus for consumer involvement in research planning (Oliver *et al*. 2004) is not likely to be problematic and that a range of stakeholders may hold compatible views on research priorities.

### Study methodology and limitations

The study sample method was one of both convenience and self-selection. Sampling using a convenience method may result in a sample that does not accurately reflect the prevalence of groups in the wider population. However, strong representation from key professional groups and consumer groups was obtained. Having consumer participants volunteer to complete the survey may have resulted in a selection bias. Sampling bias where participants have any control over whether to participate is usually assumed to result in a polarisation of opinion, as the individuals who self-select into an opinion-based study are likely to have stronger opinions, and therefore be more motivated to participate (Pagano 1992). Given the normal distribution of scores in most instances, it was concluded that this was not the case in the current study. Therefore, the self-selection of patients and carers into the study is unlikely to impact the overall conclusion of the study. The high proportion of women in the patient group suggests that the patient preferences described here may not adequately represent the views of male patients. Combining patients at various stages of care and support people groups into one weighting exercise for the research population question may have been challenging for participants. While this may have been difficult for the respondents, it does reflect the sorts of decisions that need to be made during strategic allocation of funding. A further limitation is that the reliability and validity of the scale are yet to be tested.

A further limitation that exists in the current sample relates to the lack of participants who have experienced palliative care. This means that research into psychosocial issues underlying this stage of care may have received lower priority weightings, which was certainly the case. Recent research has determined that fewer patients with haematological malignancies were involved with palliative compared with patients with colorectal cancer, and that more patients die in the acute care setting (Maddocks *et al*. 1994). A recent survey of Australian haematologists revealed that respondents did not refer patients to palliative care for a variety of reasons, which included wishing to remain optimistic, the lack of experience of palliative care services in managing haematological cancer patients and the difficulty in defining patients from this group as terminal (Auret *et al*. 2003). The lack of experience with palliative care within this patient population, and the quick deterioration that often occurs between curative treatment and death, may account for the lack of patients in the current sample who have experienced this form of care. This may in turn explain the lower priority given to examining psychosocial issues in this stage. Further research will need to address the lower referral of this patient group to palliative care, before psychosocial issues can be examined.

## CONCLUSIONS

The data suggest that a substantial proportion of psychosocial research resources for haematological cancer patients should be directed towards patients who are newly diagnosed or receiving treatment rather than later stages of the cancer journey. While there appeared to be no strong preference for prioritisation of particular types of psychosocial research, this may reflect the general need for greater research into the range of potential interventions to improve psychosocial outcomes for patients with haematological cancers. The views of various stakeholders are often compatible although not identical.
